# Long Noncoding RNA Lnc-TLN2-4:1 Suppresses Gastric Cancer Metastasis and Is Associated with Patient Survival

**DOI:** 10.1155/2020/8681361

**Published:** 2020-03-11

**Authors:** Yuyun Wu, Ningbo Hao, Suming Wang, Xin Yang, Yufeng Xiao, Huan Yang, Shiming Yang, Bosheng Li

**Affiliations:** ^1^Department of Gastroenterology, Xinqiao Hospital, Army Medical University (Third Military Medical University), Chongqing 400037, China; ^2^Department of Gastroenterology, PLA Rocket Force Characteristic Medical Center, Beijing Xinjiekouwai Street, Beijing 100088, China

## Abstract

Gastric cancer (GC) is one of the most common malignancies worldwide, and the tumor metastasis leads to poor outcomes of GC patients. Long noncoding RNAs (lncRNAs) have emerged as new regulatory molecules that play a crucial role in tumor metastasis. However, the biological function and underlying mechanism of numerous lncRNAs in GC metastasis remain largely unclear. Here, we report a novel lncRNA, lnc-TLN2-4:1, whose expression is decreased in GC tissue versus matched normal tissue, and its low expression is involved in the lymph node and distant metastases of GC, as well as poor overall survival rates of GC patients. We further found that lnc-TLN2-4:1 inhibits the ability of GC cells to migrate and invade but does not influence GC cell proliferation and confirmed that lnc-TLN2-4:1 is mainly located in the cytoplasm of GC cells. We then found that lnc-TLN2-4:1 increases the mRNA and protein expression of TLN2 in GC cells and there is a positive correlation between the expression of lnc-TLN2-4:1 and TLN2 mRNA in GC tissue. Collectively, we identified a novel lncRNA, lnc-TLN2-4:1, in GC, where lnc-TLN2-4:1 represses cell migration and invasion. The low expression of lnc-TLN2-4:1 is associated with poor overall survival rates of GC patients. These suggest that lnc-TLN2-4:1 may be a tumor suppressor during GC metastasis.

## 1. Introduction

Gastric cancer (GC) is the fifth most common cancer and the third leading cause of cancer mortality worldwide [1]. Patients with early GC who have been subject to operation have satisfactory outcome. However, for patients with advanced GC, in spite of the successful surgery and optimized chemotherapy, the survival time remains still poor [2]. The major reason that leads the patient to die is GC metastasize [3], but the underlying mechanism remains largely unclear.

Long noncoding RNAs (lncRNAs) are a class of single RNAs with more than 200 nucleotides in length and fail to encode protein [4]. In the past decade, lncRNAs have been demonstrated to play important roles in a variety of diseases, including cancer. For example, lncRNAs can affect cell proliferation, apoptosis, migration, invasion, adherence, etc, in the development of malignancy [5]. There are several regulatory mechanisms involved in lncRNAs, such as (1) lncRNAs interact with proteins, resulting the functional change of the proteins or their locations in the cell organs [6]; (2) lncRNAs serve as competitive endogenous RNAs that absorb miRNAs, thereby controlling the expression of miRNAs' target genes [7]; (3) lncRNAs also bind to mRNAs and then prevent mRNAs from degradation, or influence their translation [8]. A recent report showed that lncRNA GMAN promotes translation of ephrin A1 (EFNA1) mRNA into protein via binding to the antisense GMAN-AS, which is complementary to EFNA1 mRNA, resulting in the enhancing ability of GC cells to metastasize and invade, so that it leads to GC metastasis and poor patient survival [9]. Even so, for GC, there are numerous lncRNAs which have not been identified and their biological functions and the underlying mechanisms have not been explored yet. Interestingly, after analyses of our previous microarray data (GSE58828), we found an unidentified lncRNA, lnc-TLN2-4:1, whose expression is significantly decreased in GC tissue compared with matched normal tissue. However, the role and mechanism of this lncRNA in GC remains unknown.

Talin (TLN) plays a crucial role in cell migration, invasion, and cancer metastasis [10]. TLN gene encodes two TLN isoforms, TLN1 and TLN2. TLN2 is composed of 2532 amino acids that are 74% identical (86% similar) to human TLN1 which contains 2541 amino acids, and the complete sequencing has indicated that lower eukaryotes encode only one TLN gene corresponding to TLN1, whereas vertebrate animals possess two TLN genes [11], suggesting that TLN2 has a specific function in these species. In the past decades, a large number of studies have demonstrated the biological function of TLN1 in the development of several types of cancers [12–15], including GC [16], but there is little evidence with regard to the role of TLN2 in GC metastasis.

In the present study, we found a novel lncRNA, lnc-TLN2-4:1, located in the cytoplasm of GC cells, whose expression is decreased in GC tissue compared with matched normal tissue and is involved in poor overall survival rates of GC patients. We further found that lnc-TLN2-4:1 overexpression inhibits GC cell migration and invasion, but does not affect GC cell proliferation. These suggest that lnc-TLN2-4:1 may be a tumor suppressor during GC metastasis.

## 2. Materials and Methods

### 2.1. Patients and Specimens

Forty-nine pairs of fresh human GC samples in this study were collected from the consenting individuals based on the instructions approved by the Ethics Review Board at Xinqiao Hospital, Army Medical University (Third Medical University), from 2013 to 2017. The GC tissues were processed in the operating room and stored in liquid nitrogen within 10 min. The matched normal tissues were collected at a distance of >5 cm from the tumor tissues, and all tissues were identified histologically. None of the patients underwent chemotherapy or radiotherapy before operation. A four-year follow-up of the 49 GC patients was performed.

### 2.2. Cell Culture

Six human GC cell lines (AGS, MKN45, MGC803, BGC823, SGC7901, and MKN74) were purchased from BeNa culture Collection (BNCC). AGS cells were cultured in the F12 medium (HyClone Logan, UT, USA) supplemented with 10% FBS (Gibco BRL), and the other cells were cultured in the DMEM/HIGH GLUCOSE medium (HyClone Logan, UT, USA) supplemented with 10% FBS (Gibco BRL) at 37°C in an atmosphere of 5% CO_2_.

### 2.3. RNA Extraction, Quantitative Reverse-Transcriptase Polymerase Chain Reaction (qRT-PCR), and Immunoblotting

The procedures and reagents of RNA extraction, qRT-PCR, and immunoblotting are described in our previous study [17]. For qRT-PCR experiments, the expression of lnc-TLN2-4:1 and TLN2 was normalized to an internal control, *β*-actin, using the 2^–ΔΔCt^ method. The primer sequences are as the follows: TLN2 sense: 5′ACGGCGGAACCAGAGGAGAT3′, TLN2 antisense: 5′GGTGTCCAGGTCGGCAATGAT3′; lnc-TLN2-4:1 sense: 5′GCTGGCTGCTTCTGAGACTTAC3′, lnc-TLN2-4:1 antisense: 5′TGGAGCAACAGACTGAGGACAT3′. The parameter of PCR running is 95°C for 1 min, followed by 40 cycles of 95°C for 15 sec and 60°C for 30 sec. For immunoblotting, the anti-TLN2 antibody (ab108967) was purchased from Abcam, China (Shanghai, China), and HRP-conjugated secondary antibody was purchased from Zhongshan Biotechnology (Beijing, China), and all antibodies were used according to the manufacturer's instructions.

### 2.4. Vector and Lentivirus Construction

The LV5-V6256-1 vector containing lnc-TLN2-4:1 cDNA sequence was synthesized from a company, GenePharma (Shanghai, China). The lentivirus construction is described in our previous study [18].

### 2.5. Cell Migration, Invasion, and Proliferation

The procedures and reagents of cell migration, invasion, and proliferation experiments are described in our previous study [18]. BGC823 and SGC7901 cells which were transfected with control or lnc-TLN2-4:1-overexpressing vector were used to perform the cell migration, invasion, and proliferation experiments. The statistics of cell migration and invasion are based on three different-area images from each transwell.

### 2.6. Statistical Analysis

All data are presented as the means ± standard deviation or standard error. The difference between two groups was analyzed using Student's *t* test or Mann–Whitney *U* test. The one-way ANOVA was used to analyze the difference among three or more groups. Receiver operating characteristic curve (ROC) was used to assess the power of distinguishing two groups. The patient survival was analyzed using the Kaplan–Meier method and log-rank test. *P* < 0.05 was considered statistically significant. All statistical analyses were performed using SPSS 19.0 (Chicago, IL, USA) and GraphPad Prism 8.0 (Graphpad Software Inc, California).

## 3. Results

### 3.1. Lnc-TLN2-4:1 Expression Is Frequently Decreased in GC Tissues Compared with Matched Normal Tissues and Is Associated with GC Metastasis

**Figure 1:**
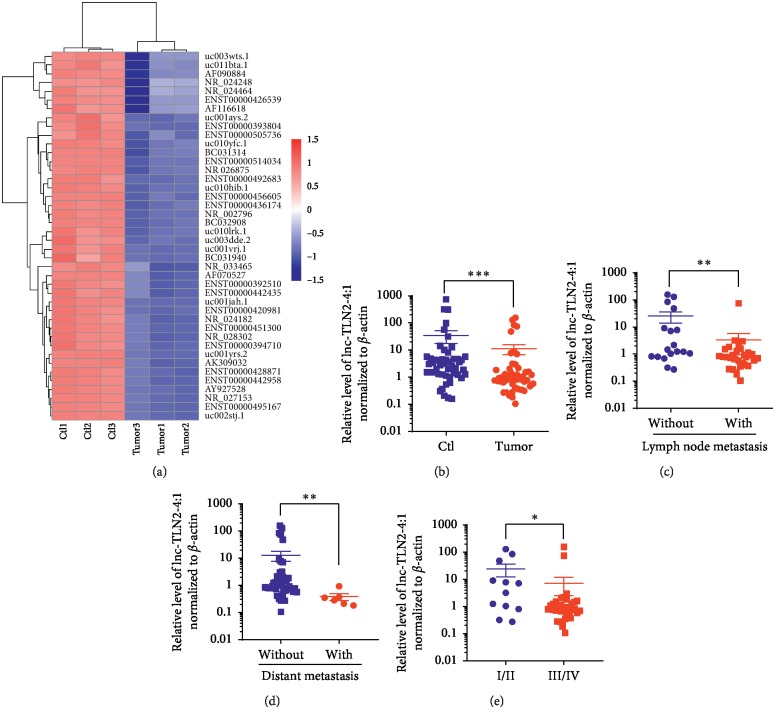
Lnc-TLN2-4:1 expression is frequently decreased in GC tissues compared with matched normal tissues and is associated with GC metastasis. (a) A heatmap shows the aberrant expression of lncRNAs in three pairs of GC and matched normal tissues detected by a Human LncRNA Microarray. (b) Scatter plots show the expression of lnc-TLN2-4:1 in 49 pairs of GC and matched normal tissues, detected by qRT-PCR, and *β*-actin serves as the internal control. (c) Scatter plots show the expression of lnc-TLN2-4:1 in 49 GC tissues, 31 of which are of lymph node metastasis and 18 of which are not. (d) Scatter plots show the expression of lnc-TLN2-4:1 in 49 GC tissues, 6 of which are of distant metastasis and 43 of which are not. (e) Scatter plots show the expression of lnc-TLN2-4:1 in 49 GC tissues, 12 of which are the sum of TNM stage I and II, and 37 of which are the sum of TNM stage III and IV. ^*∗*^*P* < 0.05, ^*∗∗*^*P* < 0.01, and ^*∗∗∗*^*P* < 0.0001.

To find a novel lncRNA which may be of regulatory function in the development of GC, we analyzed the data from an lncRNA microarray (GSE58828) that was performed in our previous study [19]. Based on stringent filtering criteria (fold change > 2, *P* < 0.01, and the lengths of lncRNAs are between 1000 nt and 2000 nt), we found an unidentified lncRNA, AF070527, whose expression was decreased in three GC tissues compared with the matched normal tissues (Figure 1(a)). The name of this lncRNA has been updated to lnc-TLN2-4:1 in LncBook, a curated knowledgebase of human lncRNAs (https://bigd.big.ac.cn/lncbook/index). Lnc-TLN2-4:1 is an intergenic lncRNA and shown to have no encoding capacity (LncBook).

To further determine the exact expression of lnc-TLN2-4:1 in GC, we collected 49 pairs of GC tissues and matched normal tissues from the enrolled GC patients. QRT-PCR experiments revealed that the expression of lnc-TLN2-4:1 is significantly decreased in GC tissue versus matched normal tissue (Figure 1(b)). We next analyzed the correlation of the clinical characteristics of GC patients with the expression of lnc-TLN2-4:1 and found that the expression of lnc-TLN2-4:1 is significantly decreased in GC tissue with lymph node metastasis, distant metastasis, or TNM stage I and II compared to that without lymph node metastasis, distant metastasis, or with TNM stage III and IV (Figures 1(c)–1(e)). However, there is no correlation of the expression of lnc-TLN2-4:1 with the patients' gender and age, as well as tumor size and differentiation (data not show). These data suggest that lnc-TLN2-4:1 is a novel lncRNA significantly decreased in GC and associated with GC metastasis.

### 3.2. Lnc-TLN2-4:1 Expression Is Associated with Overall Survival Rates of GC Patients

**Figure 2:**
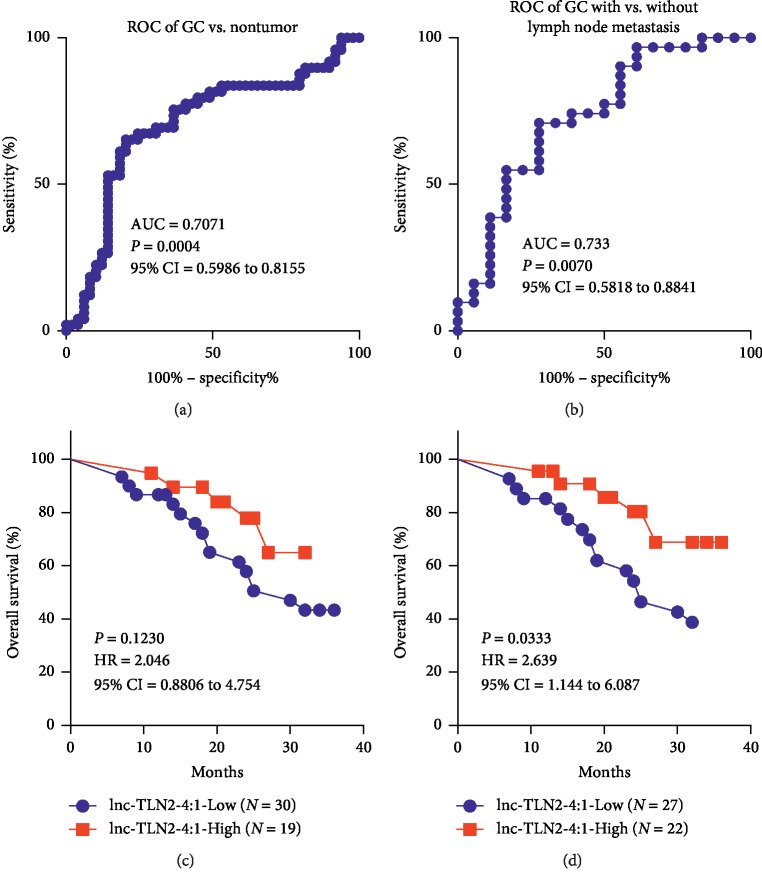
Lnc-TLN2-4:1 expression is associated with overall survival rates of GC patients. (a) ROC curve shows that the expression of lnc-TLN2 has an AUC of 0.7071 in distinguishing GC tissue from nontumor tissue, with the sensitivity of 65.31% and the specificity of 81.25% and the cutoff value of 1.246. (b) ROC curve shows that the expression of lnc-TLN2 has an AUC of 0.733 in distinguishing GC tissue from nontumor tissue, with the sensitivity of 70.97% and the specificity of 72.22% and the cutoff value of 1.016. (c) Overall survival analysis shows the survival rates of GC patients with low or high expression of lnc-TLN2-4:1, which is defined by the cutoff value of 1.246. (d) Overall survival analysis shows the survival rates of GC patients with low or high expression of lnc-TLN2-4:1, which is defined by the cutoff value of 1.016.

We next determined whether the expression of lnc-TLN2-4:1 in GC tissue has a diagnostic power for GC. Receiver operating characteristic curve (ROC) analyses revealed an AUC of 0.7071, with the sensitivity of 65.31% and the specificity of 81.25% and the cutoff value of 1.246, in discriminating GC tissues from matched normal tissues (Figure 2(a)), and an AUC of 0.733, with the sensitivity of 70.97% and the specificity of 72.22% and the cutoff value of 1.016, in distinguishing GC tissues with lymph node metastasis from those without lymph node metastasis (Figure 2(b)). We further analyzed the correlation of lnc-TLN2-4:1 expression with overall survival rates of the GC patients based on the different cutoff values obtained from the two above ROC curves and found that when the cutoff value of 1.246 was used to define the low or high expression of lnc-TLN2-4:1, there are no significant difference of overall survival rates between GC patients with low and high expression of lnc-TLN2-4:1 (Figure 2(c)). However, when the cutoff value of 1.016 was used, the significant difference of overall survival rates was observed (Figure 2(d)). These data suggest that lnc-TLN2-4:1 expression may be a prognostic marker for GC.

### 3.3. Lnc-TLN2-4:1 Represses GC Cell Migration and Invasion *In Vitro*

**Figure 3:**
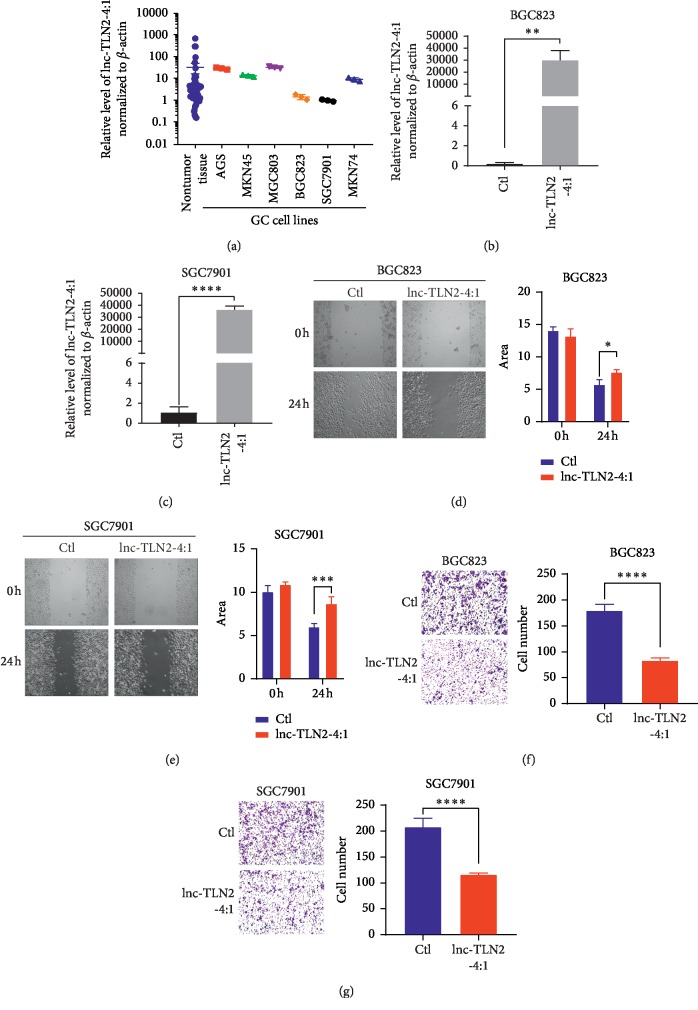
Lnc-TLN2-4:1 represses GC cell migration and invasion *in vitro*. (a) Scatter plots show the expression of lnc-TN2-4:1 in 49 nontumor tissues and GC cell lines, detected by qRT-PCR, and *β*-actin serves as the internal control. (b and c) Bars show the expression of lnc-TLN2-4:1 in BGC823 and SGC7901 cells which were transfected with lnc-TLN2-4:1-overexpressing vectors, detected by qRT-PCR, and *β*-actin serves as the internal control. (d and e) Wound healing experiments show the abilities of BGC823 and SGC7901 cells which were transfected with lnc-TLN2-4:1-overexpressing vectors to migrate. Bars show the statistics based on three independent experiments. Area indicates the area without cells in the images, calculated by Image J. (f and g) Transwell experiments show the ability of BGC823 and SGC7901 cells which were transfected with lnc-TLN2-4:1-overexpressing vectors to invasive. Bars show the statistics based on three independent experiments. ^*∗*^*P* < 0.05, ^*∗∗*^*P* < 0.01, ^*∗∗∗*^*P* < 0.001, and ^*∗∗∗∗*^*P* < 0.0001.

As abovementioned, aberrant expression of lnc-TLN2-4:1 was associated with GC metastasis; therefore, we directly investigated whether lnc-TLN2-4:1 could influence the migration and invasion of GC cells. To perform the gain-of-function, we measured the expression of lnc-TLN2-4:1 in six GC cell lines, including AGS, MKN45, MGC803, BGC823, SGC7901, and MKN74 and found that BGC823 and SGC7901 almost have the lowest expression of lnc-TLN2-4:1 (Figure 3(a)); thus, we performed the ectopic expression of lnc-TLN2-4:1 in the two GC cells using a lentivirus containing lnc-TLN2-4:1-overexpressing vectors (Figure 3(b)). Wound healing and transwell assays showed that upregulation of lnc-TLN2-4:1 significantly inhibits the migration and invasion of BGC823 and SGC7901 cells *in vitro* (Figures 3(c) and 3(d)). Because cell proliferation commonly occurred in GC development, including GC metastasis, we also determined whether lnc-TLN2-4:1 could affect GC cell proliferation. However, an assay based on a CCK-8 kit revealed that lnc-TLN2-4:1 overexpression cannot modify the proliferative ability of BGC823 and SGC7901 cells ([Supplementary-material supplementary-material-1]). These data suggest that lnc-TLN2-4:1 may be a tumor suppressor which represses GC cell metastasis but not proliferation.

### 3.4. Lnc-TLN2-4:1 Is Located in GC Cell Cytoplasm, and Its Expression Is Positively Correlated with TLN2 Expression in GC Tissues

**Figure 4:**
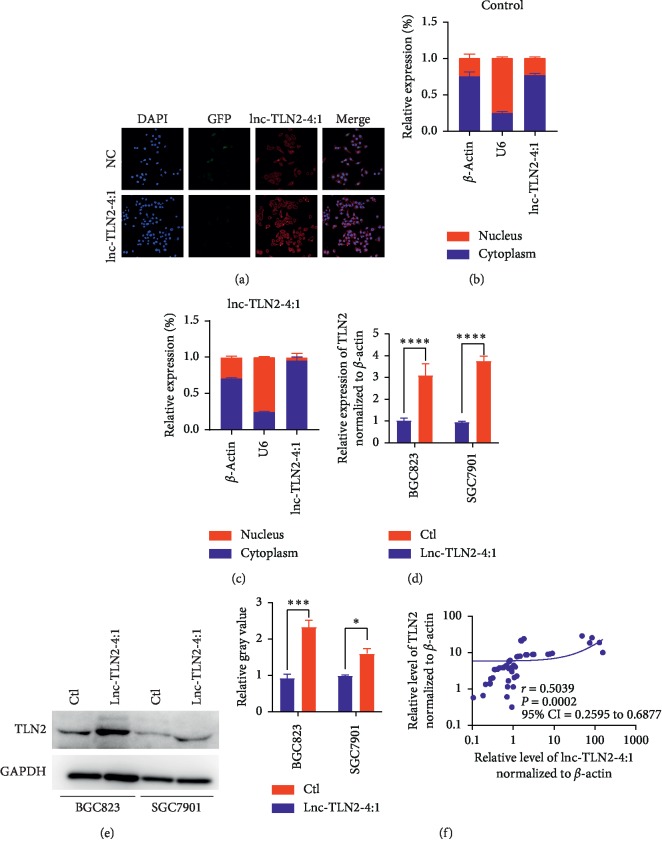
Lnc-TLN2-4:1 is located in the GC cell cytoplasm, and its expression is positively correlated with TLN2 expression in GC tissues. (a) Fluorescence in situ hybridization (FISH) shows the expression and location of lnc-TLN2-4:1 in BGC823 cells. DAPI indicates cell nucleus. GFP indicates the expression status of the vectors (negative control and lnc-TLN2-4:1-overexpressing vectors). Red fluorescence indicates the expression of lnc-TLN2-4:1. (b and c) Bars show the relative expression % of lnc-TLN2-4:1 in the cytoplasm and nucleus of BGC823 cells which were transfected with the control or lnc-TLN2-4:1-overexpressing vectors, detected by qRT-PCR, and *β*-actin serves as the internal control in the cytoplasm and U6 serves as the internal control in the nucleus. (d) Bars show the expression of TLN2 in BGC823 and SGC7901 cells which were transfected with control or lnc-TLN2-4:1-overexpressing vectors, detected by qRT-PCR, and *β*-actin serves as the internal control. (e) Western blotting shows the protein expression of TLN2 in BGC823 and SGC7901 cells which were transfected with the control or lnc-TLN2-4:1-overexpressing vectors, and GAPDH serves as the internal control. Bars show the statistics based three independent experiments. (f) The correlation between the expression of TLN2 mRNA and lnc-TLN2-4:1 in 49 pairs of GC tissues, detected by qRT-PCR, and *β*-actin serves as the internal control. ^*∗*^*P* < 0.05, ^*∗∗∗*^*P* < 0.001, and ^*∗∗∗∗*^*P* < 0.0001.

To well understand the underlying mechanism of lnc-TLN2-4:1 in GC metastasis, we determined the location of lnc-TLN2-4:1 in GC cells because the regulatory mechanism of the lncRNA is constrained by its location. We found that lnc-TLN2-4:1 is mainly located in the cytoplasm of BGC823 cells, and the expression of lnc-TLN2-4:1 is significantly increased in the cytoplasm of BGC823 cells with the ectopic expression of lnc-TLN2-4:1 compared to those with wild-type expression of lnc-TLN2-4:1 (Figure 4(a)–4(c)), suggesting that the location of the ectopic expression of lnc-TLN2-4:1 in GC cells is corresponding to its natural location and reflecting its real function. TLN2 is a coding gene which has been reported to be involved in cancer metastasis. By the nucleotide blast, we found that in lnc-TLN2-4:1 and TLN2 mRNA exist a large number of overlapped nucleotides. Therefore, we supposed whether lnc-TLN2-4:1 could regulate the expression of TLN2 mRNA in GC cells. QRT-PCR and western blotting experiments showed that lnc-TLN2-4:1 upregulation significantly increases the mRNA and protein expression of TLN2 (Figures 4(d) and 4(e)). We further analyzed the expression of lnc-TLN2-4:1 and TLN2 mRNA in GC tissues and found that there is a positive correlation between their expressions in 49 GC tissues (Figure 4(f)). These data suggest that lnc-TLN2-4:1 inhibits GC metastasis through regulating the expression of TLN2 mRNA.

## 4. Discussion

Lnc-TLN2-4:1 is predicted to have no encoding capacity and has 1558 nt in length. In this study, we found that lnc-TLN2-4:1 expression is significantly decreased in GC tissue versus matched normal tissue and is associated with the GC cell lymph node and distant metastases. ROC analyses revealed that lnc-TLN2-4:1 expression has a potentially predictive power in distinguishing GC tissue from matched normal tissue, and the decreased expression of lnc-TLN2-4:1 is closely involved in poor overall survival rates of GC patients. So far, there are hundreds of lncRNAs that have been identified to have aberrant expression in GC development and also be considered as potential biomarkers for GC detection. For example, Zhuo et al. report an lncRNA, GMAN, which is overexpressed in GC tissue versus nontumor tissue and its upregulation is also associated with poor overall survival rates of GC patients [9]; Zhang et al. report an lncRNA, HOXC-AS3, whose expression is increased in GC tissue versus nontumor tissue and correlated with clinical outcomes of GC [20]. These instances suggest that lncRNAs may be a potential biomarker for GC detection, and our findings also suggest that lnc-TLN2-4:1 may be a novel biomarker for the diagnosis and prognosis of GC and hint that it may have an important role during GC metastasis.

To determine the biological function of lnc-TLN2-4:1 in GC, we selected two GC cell lines which have low expression of lnc-TLN2-4:1 and constructed BGC823 and SGC7901 cells with stably ectopic expression of lnc-TLN2-4:1 using a lentivirus containing lnc-TLN2-4:1-overexpressing vectors. This effect of ectopic expression of lnc-TLN2-4:1 was identified by qRT-PCR and immunofluorescence. Stably modified expression of lncRNAs using lentivirus is widely used in studying their biological functions, such as in our previous study [18]. The biological functions of lncRNAs are strongly associated with their location in cells, and the results of immunofluorescence experiments in our study indicate that the location of lnc-TLN2-4:1 with ectopic expression is corresponding to its natural location. Our findings revealed that lnc-TLN2-4:1 upregulation can significantly inhibit the migration and invasion of GC cells but does not affect GC cell proliferation, suggesting that lnc-TLN2-4:1 acts as a tumor suppressor in GC metastasis.

To address the underlying mechanism by which lnc-TLN2-4:1 represses GC metastasis, we searched for the candidate target genes of lnc-TLN2-4:1. Because lnc-TLN2-4:1 is located in the cell cytoplasm, we considered that lnc-TLN2-4:1 has a possibility of regulating TLN2 mRNA stability. A large number of studies have reported that lncRNAs can protect mRNAs from degradation [21, 22]. The specific stability effect of lncRNAs on mRNAs is based on the complementary base pairing, and our findings revealed that the nucleotide sequence of lnc-TLN2-4:1 completely overlaps the 3′end fragment of TLN2. Our further investigation showed that lnc-TLN2-4:1 upregulation increases the mRNA expression of TLN2 in GC cells and there is a positive correlation between the expression of lnc-TLN2-4:1 and TLN2 mRNA in 49 GC tissues. These data suggest that lnc-TLN2-4:1 inhibits GC metastasis through regulating the expression of TLN2 mRNA, but the underlying mechanism needs to be identified in the future.

In conclusion, we identified a novel lncRNA, lnc-TLN2-4:1, which is downregulated in GC tissue versus matched normal tissue and whose low expression is associated with GC metastasis and poor overall survival rates of GC patients. We further found that lnc-TLN2-4:1 represses the ability of GC cells to migrate and invade, and lnc-TLN2-4:1 promotes the expression of TLN2 in GC cells and there is a positive correlation between the expression of lnc-TLN2-4:1 and TLN2 in GC tissues. These data suggest that lnc-TLN2-4:1 may be a therapeutic target for GC.

## Data Availability

All data generated or analyzed during this study are included in this published article (and its supplementary information files), and more detailed data are available from the corresponding author on reasonable request.

## Supplementary Materials

Supplementary Materials Figure S1. Lnc-TLN2-4:1 doses not affect the abilities of BGC823 and SGC7901 cells to proliferate. (A and B) the proliferative abilities of BGC823 and SGC7901 cells which were transfected with the control or lnc-TLN2-4:1-overexpressing vectors were analyzed using a CCK-8 kit.

## References

[1] Bray F., Ferlay J., Soerjomataram I., Siegel R. L., Torre L. A., Jemal A. (2018). Global cancer statistics 2018: GLOBOCAN estimates of incidence and mortality worldwide for 36 cancers in 185 countries. *CA: A Cancer Journal for Clinicians*.

[2] Salati M., Orsi G., Smyth E. (2019). Gastric cancer: translating novels concepts into clinical practice. *Cancer Treatment Reviews*.

[3] Alyami M., Hübner M., Grass F. (2019). Pressurised intraperitoneal aerosol chemotherapy: rationale, evidence, and potential indications. *The Lancet Oncology*.

[4] Zeng S., Xiao Y. F., Tang B. (2015). Long noncoding RNA in digestive tract cancers: function, mechanism, and potential biomarker. *The Oncologist*.

[5] Kopp F., Mendell J. T. (2018). Functional classification and experimental dissection of long noncoding RNAs. *Cell*.

[6] Liu B., Sun L., Liu Q. (2015). A cytoplasmic NF-*κ*B interacting long noncoding RNA blocks I*κ*B phosphorylation and suppresses breast cancer metastasis. *Cancer Cell*.

[7] Hu W. L., Jin L., Xu A. (2018). GUARDIN is a p53-responsive long non-coding RNA that is essential for genomic stability. *Nature Cell Biology*.

[8] Yuan J.-h., Yang F., Wang F. (2014). A long noncoding RNA activated by TGF-*β* promotes the invasion-metastasis cascade in hepatocellular carcinoma. *Cancer Cell*.

[9] Zhuo W., Liu Y., Li S. (2018). Long non-coding RNA GMAN, upregulated in gastric cancer tissues, is associated with metastasis in patients and promotes translation of ephrin A1 by competitively binding GMAN-AS. *Gastroenterology*.

[10] Qi L., Jafari N., Li X. (2016). Talin2-mediated traction force drives matrix degradation and cell invasion. *Journal of Cell Science*.

[11] Monkley S. J., Pritchard C. A., Critchley D. R. (2001). Analysis of the mammalian talin2 gene TLN2. *Biochemical and Biophysical Research Communications*.

[12] Jin J.-K., Tien P.-C., Cheng C.-J. (2015). Talin1 phosphorylation activates *β*1 integrins: a novel mechanism to promote prostate cancer bone metastasis. *Oncogene*.

[13] Lai M.-T., Hua C.-H., Tsai M.-H. (2011). Talin-1 overexpression defines high risk for aggressive oral squamous cell carcinoma and promotes cancer metastasis. *The Journal of Pathology*.

[14] Singel S. M., Cornelius C., Batten K. (2013). A targeted RNAi screen of the breast cancer genome identifies KIF14 and TLN1 as genes that modulate docetaxel chemosensitivity in triple-negative breast cancer. *Clinical Cancer Research*.

[15] Sakamoto S., McCann R. O., Dhir R., Kyprianou N. (2010). Talin1 promotes tumor invasion and metastasis via focal adhesion signaling and anoikis resistance. *Cancer Research*.

[16] Li W. Q., Hu N., Wang Z. (2013). Genetic variants in epidermal growth factor receptor pathway genes and risk of esophageal squamous cell carcinoma and gastric cancer in a Chinese population. *PLoS One*.

[17] Li B.-S., Zuo Q.-F., Zhao Y.-L. (2015). MicroRNA-25 promotes gastric cancer migration, invasion and proliferation by directly targeting transducer of ERBB2, 1 and correlates with poor survival. *Oncogene*.

[18] Zeng S., Xie X., Xiao Y. F. (2017). Long noncoding RNA LINC00675 enhances phosphorylation of vimentin on Ser83 to suppress gastric cancer progression. *Cancer Letters*.

[19] Lu M. H., Tang B., Zeng S. (2015). Long noncoding RNA BC032469, a novel competing endogenous RNA, upregulates hTERT expression by sponging miR-1207-5p and promotes proliferation in gastric cancer. *Oncogene*.

[20] Zhang E., He X., Zhang C. (2018). A novel long noncoding RNA HOXC-AS3 mediates tumorigenesis of gastric cancer by binding to YBX1. *Genome Biology*.

[21] Gong C., Maquat L. E. (2011). lncRNAs transactivate STAU1-mediated mRNA decay by duplexing with 3′ UTRs via Alu elements. *Nature*.

[22] Yuan J.-h., Liu X.-n., Wang T.-t. (2017). The MBNL3 splicing factor promotes hepatocellular carcinoma by increasing PXN expression through the alternative splicing of lncRNA-PXN-AS1. *Nature Cell Biology*.

