# Supplementary material for: Long Noncoding RNA Lnc-TLN2-4:1 Suppresses Gastric Cancer Metastasis and Is Associated with Patient Survival
Source: J Oncol. 2020 Mar 11;2020:8681361. doi: 10.1155/2020/8681361 (PMC7086451; doi:10.1155/2020/8681361)
Supplement: Supplementary Materials — Figure S1. Lnc-TLN2-4:1 doses not affect the abilities of BGC823 and SGC7901 cells to proliferate. (A and B) the proliferative abilities of BGC823 and SGC7901 cells which were transfected with the control or lnc-TLN2-4:1-overexpressing vectors were analyzed using a CCK-8 kit. [file 8681361.f1.pdf]

SUPPLEMENTARY FIGURE LEGENDS

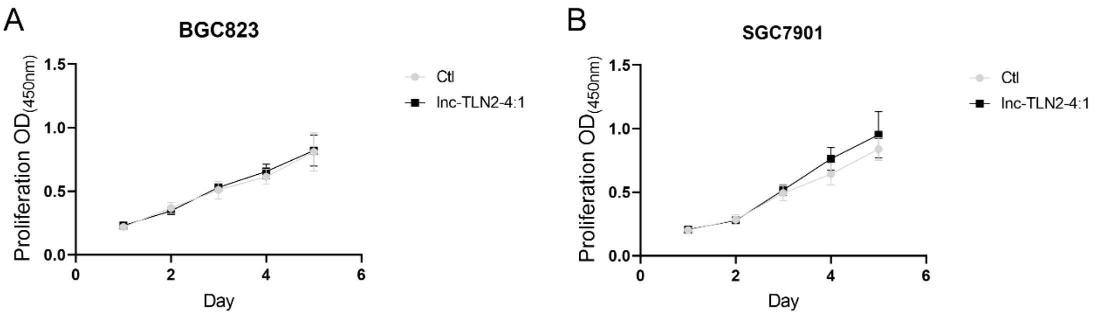

Figure S1.lnc-TLN2-4:1 doses not affect the abilities of BGC823 and SGC7901 cells to proliferate. **(A and B)** the proliferative abilities of BGC823 and SGC7901 cells which were transfected with control or lnc-TLN2-4:1-overexpressing vectors were analyzed using a CCK8 kit.
